# Gesundheitsinformationsverhalten 65+: Erreichbarkeit älterer Zielgruppen

**DOI:** 10.1007/s00103-020-03253-1

**Published:** 2020-12-01

**Authors:** Paula Stehr, Winja Weber, Constanze Rossmann

**Affiliations:** Seminar für Medien- und Kommunikationswissenschaft, Universität Erfurt, Nordhäuser Str. 63, 99089 Erfurt, Deutschland

**Keywords:** Ältere und Hochaltrige, Gesundheitsinformationsverhalten, Theorie- und evidenzbasierte Gesundheitskommunikation, Erreichbarkeitsplanung, Zielgruppensegmentierung, Older adults, Health information behavior, Theory- and evidence-based communication, Media strategy, Audience segmentation

## Abstract

**Hintergrund:**

Der Anteil älterer Personen in der Bevölkerung wächst stetig. Gleichzeitig steigen im Alter die Risiken für gesundheitliche Probleme. Aus Sicht der Gesundheitsförderung ist es deshalb wichtig, diese Zielgruppe zu erreichen. Für die Auswahl geeigneter Kommunikationskanäle sollte das Mediennutzungs- bzw. Gesundheitsinformationsverhalten berücksichtigt werden. Dabei kann auch eine Segmentierung der heterogenen Zielgruppe 65+ in Teilzielgruppen notwendig sein.

**Ziel des Beitrags:**

Der Beitrag liefert aktuelle empirische Daten zum Gesundheitsinformationsverhalten der Zielgruppe 65+ insgesamt sowie spezifischer Teilzielgruppen. Auf dieser Basis können geeignete Kommunikationskanäle ausgewählt werden, um diese Gruppen gezielt zu erreichen.

**Methodik:**

Es wurden zunächst 20 Personen mit qualitativen Leitfadeninterviews befragt und anschließend eine standardisierte telefonische Befragung von 1001 zufällig ausgewählten Personen ab 65 Jahren durchgeführt.

**Ergebnisse:**

Die Zielgruppe 65+ kann am besten über interpersonale und traditionelle, massenmediale Quellen erreicht werden. Dabei spielen, insbesondere für chronisch kranke Menschen, auch gesundheitsspezifische Angebote wie Apothekenzeitschriften eine wichtige Rolle. Weiterhin stellen Gespräche mit medizinischem Personal eine wichtige Quelle für chronisch Erkrankte und Menschen mit einem eher negativen Altersbild dar. Über das Internet werden eher Männer und Personen mit höherem Einkommen erreicht.

**Diskussion:**

Die Älteren in Deutschland sind eine heterogene Zielgruppe. Bei der Erreichbarkeitsplanung müssen Unterschiede im Gesundheitsinformationsverhalten verschiedener Teilzielgruppen berücksichtigt werden. Weiterhin sollten auch Inhalte und Gestaltung von Kommunikationsmaßnahmen evidenzbasiert konzeptioniert werden.

## Abstract (en)

**Background:**

The proportion of older adults among the population is constantly growing. At the same time, the risk of health problems increases with age. From a health promotion perspective, it is therefore important to reach this target group. The selection of media and communication channels should be based on media use and health information behavior of the target group. In this context, it may be necessary to segment the heterogeneous group of older adults into subgroups.

**Objectives:**

The article provides current empirical data on the health information behavior of older adults in general and in particular subgroups. On this basis, adequate communication channels can be selected to address these groups.

**Methods:**

First, 20 people participated in guided qualitative interviews. Second, a subsequent standardized telephone survey of 1001 randomly selected people aged 65 years and above was conducted.

**Results:**

Older adults can best be reached via interpersonal and traditional mass media sources. Health-related media such as pharmacy magazines also play an important role, especially for people with chronic diseases. Furthermore, health professionals are also an important source of information for older adults with chronic diseases and a negative attitude towards aging. On the internet, men and people with a higher income are more likely to be reached.

**Conclusions:**

Older adults in Germany are a heterogeneous target group. To reach them effectively, differences in the health information behavior of subtarget groups must be considered. Furthermore, the content and design of communication measures should also be evidence based.

## Hintergrund

Eine steigende Lebenserwartung und ein Rückgang der Geburtenrate resultieren in einer Zunahme des Durchschnittsalters in der Bevölkerung. Weltweit waren im Jahr 2019 ca. 703 Mio. Menschen 65 Jahre alt oder älter, wobei Forscherinnen und Forscher eine Verdoppelung dieser Zahl bis 2050 erwarten [1]. Auch in Deutschland ist die Verschiebung der Altersstruktur deutlich sichtbar: Im Jahr 2018 lag der Bevölkerungsanteil der ab 67-Jährigen bei 19 %, wohingegen im Jahr 2060 ein Anteil dieser Altersgruppe von 24–30 % erwartet wird [2]. Durch die Alterung der Bevölkerung wird auch eine starke Zunahme von altersbedingten und chronischen Krankheiten in Deutschland prognostiziert, die zu einem erhöhten Bedarf an medizinischer Versorgung führt [3–5]. Für ältere Menschen spielt es daher eine zentrale Rolle, die Gesundheit zu erhalten, Krankheiten zu bewältigen und erforderliche gesundheitsrelevante Entscheidungen zu treffen [6].

Um ältere Menschen bestmöglich in ihrem individuellen Gesundheitsverhalten zu unterstützen, ist es wichtig, sie effektiv über Gesundheitsfragen zu informieren – insbesondere diejenigen, die von einem schlechteren Gesundheitszustand (z. B. chronischen Erkrankungen) betroffen und deshalb besonders auf Gesundheitsinformationen angewiesen sind. Da dies jedoch nicht auf einer breiten Basis im Rahmen der Gesundheitsvorsorge geleistet werden kann, spielen mediale Kommunikationsangebote (z. B. im Rahmen von Kampagnen) dabei eine zentrale Rolle. Welche Kommunikationskanäle jedoch genau für die Zielgruppe geeignet sind, sollte zunächst auf Basis empirischer Evidenzen ermittelt werden. So ist basierend auf dem Rahmenmodell der theorie- und evidenzbasierten Kampagnenplanung [7] zum einen die Auswahl von Inhalten und Botschaften sowie deren Aufbereitung für eine effektive Kommunikationsstrategie entscheidend. Zum anderen spielt auch die Auswahl geeigneter Kommunikationswege und Kanäle auf Basis von Erkenntnissen zum Mediennutzungs- bzw. Gesundheitsinformationsverhalten der Zielgruppe eine wichtige Rolle. Ältere Personen unterscheiden sich in ihrem allgemeinen Mediennutzungs- sowie ihrem Gesundheitsinformationsverhalten jedoch deutlich von jüngeren Zielgruppen [8, 9], weshalb das Gesundheitsinformationsverhalten der ab 65-Jährigen im vorliegenden Beitrag genauer beschrieben werden soll.

Zudem muss berücksichtigt werden, dass ältere Menschen eine sehr heterogene Zielgruppe darstellen [10]. Der Heterogenität von Bevölkerungsgruppen wird bei der Planung von Gesundheitskampagnen Rechnung getragen, indem die Zielgruppe in mehrere Teilzielgruppen unterteilt wird [11]. Entsprechend sollte sich auch die Auswahl von Kommunikationskanälen am Gesundheitsinformationsverhalten der verschiedenen Teilzielgruppen innerhalb der Altersgruppe 65+ orientieren. Um diese Teilzielgruppen zu bestimmen, werden neben Merkmalen, die mit dem Zielverhalten in Verbindung stehen (z. B. Gesundheitszustand), insbesondere soziodemografische Daten (z. B. sozioökonomischer Status, Geschlecht, Alter) als Faktoren herangezogen [12, 13].

Eine wichtige Zielgruppe bei älteren Menschen stellen Personen mit gesundheitlichen Problemen wie chronischen Erkrankungen dar. Es ist wichtig, diese gezielt und effektiv über Gesundheitsfragen zu informieren. Studien zeigen, dass es einen Zusammenhang zwischen dem Gesundheitszustand und dem Gesundheitsinformationsverhalten älterer Menschen gibt. Manche deuten hierbei darauf hin, dass Personen mit gesundheitlichen Problemen – insbesondere in Form chronischer Erkrankungen – mehr nach Gesundheitsinformationen suchen [14–16], während andere zeigen, dass Personen mit einem guten Gesundheitszustand häufiger Gesundheitsinformationen nutzen [12, 17].

Personen mit einem niedrigen sozioökonomischen Status leiden weitaus häufiger an chronischen Erkrankungen und Beschwerden [18]. Entsprechende Unterschiede zeigen sich auch mit Blick auf den subjektiven Gesundheitszustand: Eine Umfrage zeigt, dass 52 % der 65- bis 85-Jährigen mit einem höheren sozioökonomischen Status ihren Gesundheitszustand positiv bewerten, während dies bei Personen mit einem niedrigen sozioökonomischen Status nur bei 28 % der Fall ist [19]. Zudem gehen Personen mit einem höheren sozioökonomischen Status stärker davon aus, einen Einfluss auf die eigene Gesundheit zu haben, als Personen mit einem niedrigen sozioökonomischen Status [19]. Dies bedeutet, dass vor allem Ältere mit einem niedrigen sozioökonomischen Status auf Unterstützung durch Informationen angewiesen sind und spezifisch durch Kanäle erreicht werden sollten, die sie besonders häufig nutzen.

Auch das Geschlecht spielt in Bezug auf das Thema Gesundheit eine Rolle. Aufgrund der höheren Lebenserwartung sind ältere Frauen häufiger als ältere Männer von Krankheiten betroffen [20]. Männer schätzen ihren Gesundheitszustand etwas positiver ein als Frauen [21], sind jedoch auch weniger gesundheitsbewusst und ergreifen seltener Präventionsmaßnahmen [22]. Deshalb müssen sowohl Männer als auch Frauen mit den für sie relevanten Gesundheitsthemen über die von ihnen genutzten Kommunikationskanäle gezielt adressiert werden.

Als weiteres soziodemografisches Merkmal könnte auch das Alter herangezogen werden. Jedoch zeigen Studien aus dem Bereich der Gerontologie, dass Menschen sehr unterschiedlich altern und das Verhalten von Seniorinnen und Senioren nicht hauptsächlich durch deren Anzahl an Lebensjahren erklärt werden kann [10, 23, 24]. Wichtiger als das chronologische Alter ist vielmehr die individuelle Wahrnehmung des Alters bzw. des Älterwerdens. Diese Wahrnehmung hängt stark mit der Denkweise und Aktivität einer Person zusammen [23]. Während einige das Älterwerden als Gewinn sehen, nehmen andere es eher als Verlust wahr [25]. Zum Beispiel kann eine 65-jährige Person noch sehr aktiv sein und den Eintritt ins Rentenalter als neuen Start sehen, während eine andere gleichaltrige Person einen sehr inaktiven Lebensstil hat und das Rentenalter als Beginn der letzten Lebensphase sieht [26]. Menschen mit einem negativen Altersbild führen Krankheitssymptome und körperliche Einschränkungen häufiger auf das Altern an sich zurück als auf behandelbare Krankheiten und zeigen deshalb ein ungünstigeres Gesundheitsverhalten [27]. Es ist deshalb wichtig zu wissen, über welche Kanäle sich Menschen mit einem eher negativen Altersbild zum Thema Gesundheit informieren, um hierüber Botschaften zu kommunizieren, die diese Vorstellungen adressieren.

Wie diese Ausführungen zeigen, ist es notwendig, die Zielgruppe 65+ als heterogene Zielgruppe zu betrachten. Um Gesundheitsinformationen gezielt an bestimmte Teilgruppen streuen zu können, müssen die Kommunikationskanäle entsprechend ausgewählt werden. Ziel des vorliegenden Beitrags ist es daher, das Gesundheitsinformationsverhalten der Älteren in Deutschland sowohl insgesamt als auch für relevante Teilzielgruppen (segmentiert nach chronischen Erkrankungen, Einkommen als Indikator des sozioökonomischen Status, Geschlecht und Altersbild) innerhalb der älteren Bevölkerung zu beschreiben. Die Basis hierfür bilden Daten aus einer zweiteiligen Studie, die im Rahmen eines Projekts zur Förderung körperlicher Aktivität^1^ erhoben wurden. Um zunächst die Bandbreite möglicher Gesundheitsinformationsquellen der Zielgruppe aufzudecken und anschließend die am häufigsten genutzten Quellen zu quantifizieren, wurden qualitative Interviews mit einer standardisierten Befragung kombiniert.

## Studie 1: Qualitative Leitfadeninterviews

### Methode

In den qualitativen Leitfadeninterviews wurden je 10 Personen in Thüringen und Nordrhein-Westfalen befragt. Unter den 20 Befragten waren gleich viele Männer und Frauen sowie Personen aus zwei Altersgruppen (65 bis 79 Jahre und ab 80 Jahren). Um eine möglichst große Heterogenität der Stichprobe zu gewährleisten, wurden bewusst auch Personen mit Migrationshintergrund und niedrigem sozioökonomischen Status rekrutiert, da diese mit Gesundheitskampagnen häufig schwerer zu erreichen sind. Die Rekrutierung erfolgte über Dritte, sodass sich die Interviewerinnen und Befragten nicht persönlich kannten. Die Interviews fanden im Juli 2018 statt und dauerten zwischen 30 und 60 min. Die Teilnehmenden erhielten eine Aufwandsentschädigung von 20 €. Sie wurden vor dem Interview über die Freiwilligkeit der Teilnahme und das Ziel der Studie aufgeklärt und willigten schriftlich in die Speicherung und Verarbeitung ihrer Daten ein. Die Wahrung ethischer Standards wurde durch eine Prüfung des Ethikbeirats der Universität Erfurt sichergestellt. Die Interviews wurden aufgezeichnet und anschließend mit dem Programm MAXQDA [29] verschriftlicht. Bei der Transkription wurden Namen und Ortsbezeichnungen durch allgemeine Beschreibungen ersetzt und jedem Teilnehmenden ein Pseudonym zugeordnet. Die Auswertung der Interviews erfolgte mittels einer inhaltlich strukturierenden Inhaltsanalyse [30].

### Ergebnisse

Die Ergebnisse der qualitativen Interviews deuten darauf hin, dass die Älteren zwar am Thema Gesundheit interessiert sind, einige sich jedoch eher ungerichtet informieren, anstatt gezielt nach Informationen zu suchen: „Ja was man halt so mitkriegt so aus den Medien und so weiter“ (Christine, 68 Jahre). Um sich allgemein zu informieren, greifen sie auf die klassischen Massenmedien wie Fernsehen, Tageszeitung und Zeitschriften zurück. Als einzelne Angebote können hierbei die *Apotheken Umschau*, Zeitschriften der Krankenkassen und Gesundheitsmagazine auf den dritten Programmen (regionale Fernsehsender) herausgegriffen werden. Dabei wurden in den qualitativen Interviews die NDR-Sendung *Visite* und die MDR-Sendung *Hauptsache Gesund* mehrfach explizit genannt. Hierbei wurde positiv hervorgehoben, dass dort über Themen informiert wird, die für breite Teile der Bevölkerung relevant sind: „Was wir gerne sehen, Sendungen vom MDR. Da ist ja Donnerstag immer ‚Gesund bleiben‘, die verfolgen wir recht gerne. Weil es eigentlich allgemeine Themen sind und, sagen wir mal, nicht so sehr spezifisch“ (Frida, 67 Jahre). Aber auch spezifische Fachinformationen können eine Rolle für Ältere spielen: „Gesundheitliche Themen finde ich mehr in spezielle^2^ Literatur. Das, was … die Zeitung schreibt, das schreiben nicht die Spezialisten … Und der spezielle Literatur, da schreiben auch Spezialisten und … die Genauigkeit ist mehr da für mich angepasst“ (Evgeny, 70 Jahre). Für die gezielte Suche nach Informationen bietet das Internet Vorteile: „Also ich bin sehr oft im Internet unterwegs, … weil da ist es am leichtesten zu suchen. Da muss man nicht jetzt losgehen und Bücher durchstöbern oder Zeitschriften“ (Jochen, 70 Jahre). Weiterhin tauschen sich Ältere mit anderen Personen über das Thema Gesundheit aus, sowohl mit medizinischem Personal als auch mit der Familie, Bekannten oder Menschen mit der gleichen Erkrankung. Die Ergebnisse zeigen, dass in Bezug auf das Gesundheitsinformationsverhalten der Zielgruppe 65+ eine große Anzahl an unterschiedlichen, potenziellen Quellen berücksichtigt werden muss. Die qualitativen Interviews machen dabei deutlich, dass die Funktionen, die Ältere diesen Quellen zuschreiben, variieren. Sowohl allgemeine massenmediale Inhalte als auch gesundheitsbezogene Rundfunk- und Printangebote dienen dazu, sich über Gesundheit zu informieren, ohne dies auf bestimmte Themen oder Erkrankungen einzugrenzen. Für spezifische Informationen wird laut Aussagen der Befragten hingegen auf Fachveröffentlichungen, das Internet und den Austausch mit medizinischem Personal oder anderen Betroffenen zurückgegriffen.

## Studie 2: Repräsentative Telefonbefragung

### Methode

Im Anschluss an die qualitative Studie wurde eine standardisierte, computergestützte Telefonbefragung durchgeführt (CATI), um die quantitative Bedeutung der unterschiedlichen Gesundheitsinformationsquellen zu analysieren. Die Grundgesamtheit umfasst Menschen ab 65 Jahren in Deutschland. Die Auswahl der Befragten erfolgte über eine zufällige Auswahl von deutschlandweiten Festnetz- und Handynummern. Die Teilnahme war freiwillig und wurde nicht vergütet. Die Daten wurden anonymisiert erhoben und das Vorgehen durch den Ethikbeirat der Universität Erfurt als unbedenklich eingestuft. Lebte mehr als eine Person ab 65 Jahren in einem Haushalt, wurde die zu befragende Person über die Next-Birthday-Methode bestimmt; also diejenige Person ab 65 Jahren befragt, die als Nächste Geburtstag hat.

### Operationalisierung

Um das Gesundheitsinformationsverhalten zu erfassen, wurde die Nutzungshäufigkeit der folgenden Quellen erfasst, die auf Basis der Leitfadeninterviews identifiziert worden waren: Gespräche mit medizinischem Personal; Gespräche mit Apotheker/innen; kostenlose Broschüren; Krankenkassenzeitschriften; Apothekenzeitschriften; Gespräche mit Familie, Freund/innen, Bekannten; Fernseh- und Radiosendungen; Gesundheitsprogramme auf regionalen Sendern; Tageszeitung oder Zeitschriften; Internet; Bücher; Veranstaltungen. Die Reihenfolge der Quellen wurde zufällig rotiert. Die Befragten konnten ihre Antwort auf einer Skala abstufen, deren Skalenpunkte für die telefonische Befragung verbalisiert wurden (1 „nie“, 2 „selten“, 3 „hin und wieder“, 4 „häufig“, 5 „sehr häufig“). Zusätzlich wurde das allgemeine Interesse an Gesundheit und Krankheitsprävention durch die Zustimmung (1 „trifft gar nicht zu“, 2 „trifft eher nicht zu“, 3 „teils, teils“, 4 „trifft eher zu“, 5 „trifft voll und ganz zu“) zu zwei Items abgefragt: „Ich bin sehr darauf bedacht, durch mein Verhalten und meine Lebensweise Krankheiten vorzubeugen“ und „Ich interessiere mich generell sehr für das Thema Gesundheit“.

Das Vorhandensein chronischer Erkrankungen wurde mit der folgenden Frage erhoben: „Haben Sie eine chronische Krankheit?“ Als Indikator für den sozioökonomischen Status wurde lediglich das Einkommen herangezogen. Während hierfür üblicherweise häufig auch der berufliche Status herangezogen wird, ist dieser bei Älteren kein geeigneter Indikator, da die meisten dieser Altersgruppe nicht mehr berufstätig sind [31] – so auch 90 % der Befragten dieser Studie. Bezüglich der formalen Bildung zeigten die Leitfadeninterviews zudem, dass die Bildungsbiografie älterer Personen mitunter durch geschichtliche Ereignisse, wie beispielsweise den Zweiten Weltkrieg, unterbrochen wurde und der formale Schulabschluss in der Jugend nicht immer aussagekräftig für den Status im Alter ist. Das Einkommen wurde als monatliches Nettohaushaltseinkommen kategorial erhoben (1 „weniger als 1000 €“, 2 „1000 bis unter 2000 €“, 3 „2000 bis unter 3000 €“, 4 „3000 bis unter 4000 €“, 5 „4000 € und mehr“) und für die Auswertung in jene mit einem eher unterdurchschnittlichen Haushaltseinkommen und jene mit einem eher durchschnittlichen oder überdurchschnittlichen Einkommen dichotomisiert. Basierend auf dem durchschnittlichen monatlichen Nettohaushaltseinkommen von Rentner/innenhaushalten wurde die Grenze hierfür bei 2000 € festgesetzt [32].

Als relevante Dimensionen des subjektiven Altersbildes wurden auf Basis der qualitativen Leitfadeninterviews verschiedene Aspekte identifiziert: körperlicher Verlust, sozialer Verlust, persönliche Weiterentwicklung, Selbstkenntnis und die Einschätzung des zukünftigen Lebens. Um für die standardisierte Befragung bereits validierte Items verwenden zu können, wurde für die Operationalisierung auf Items aus dem Deutschen Alterssurvey zurückgegriffen [33]. Die Reihenfolge der insgesamt zehn Items wurde zufällig rotiert und die Zustimmung auf einer 5‑stufigen Antwortskala erfasst (1 „trifft gar nicht zu“, 2 „trifft eher nicht zu“, 3 „teils, teils“, 4 „trifft eher zu“, 5 „trifft voll und ganz zu“). Beispielitems sind: „Ich freue mich auf das Leben, das noch vor mir liegt“ (Einschätzung des zukünftigen Lebens), „Älterwerden bedeutet für mich, dass ich körperliche Einbußen schlechter ausgleichen kann“ (körperlicher Verlust), „Älterwerden bedeutet für mich, dass ich mich häufiger einsam fühle“ (sozialer Verlust), „Älterwerden bedeutet für mich, dass sich meine Fähigkeiten erweitern“ (persönliche Weiterentwicklung) und „Älterwerden bedeutet für mich, dass ich mich selbst genauer kennen und besser einschätzen lerne“ (Selbstkenntnis). Aus den zehn Items wurde ein Index berechnet (*M* = 3,9; *SD* = 0,6; α = 0,74). Der Median lag ebenfalls bei 3,9, sodass dieser Wert als Cut-off-Kriterium gewählt wurde, um zwei gleich große Gruppen miteinander vergleichen zu können. Befragte mit einem Wert bis einschließlich 3,9 wurden der Gruppe „eher negatives Altersbild“ zugeordnet, jene mit einem Wert über 3,9 der Gruppe „eher positives Altersbild“. Die Befragten in dieser Gruppe haben also den Items überwiegend zugestimmt, da ein Wert von 4 „trifft eher zu“ bedeutete.

### Stichprobe

Insgesamt haben 1001 Personen vollständig an der durchschnittlich 30-minütigen Befragung teilgenommen. Hinsichtlich Alter und Geschlecht entspricht die Verteilung in der Stichprobe der Grundgesamtheit [34]. So waren 24 % der Befragten 80 Jahre alt oder älter und 49 % der Befragten waren männlich. In der Stichprobe waren jedoch mehr Menschen mit höherem Bildungsgrad (49 % mit (Fach‑)Hochschulreife) vertreten als in der Grundgesamtheit. Für die Darstellung von Mittelwerten und Mittelwertsunterschieden (t-Tests) wurden die Daten deshalb auf der Basis von Bundesland, Alter, Geschlecht und Bildungsabschluss gewichtet. Grundsätzlich deuten die Daten darauf hin, dass Ältere ein hohes Interesse an Gesundheit haben (*M* = 3,8; *SD* = 1,2) und darauf bedacht sind, durch ihre Lebensweise Krankheiten vorzubeugen (*M* = 4,3; *SD* = 1,0).

### Ergebnisse

**Figure 1:**
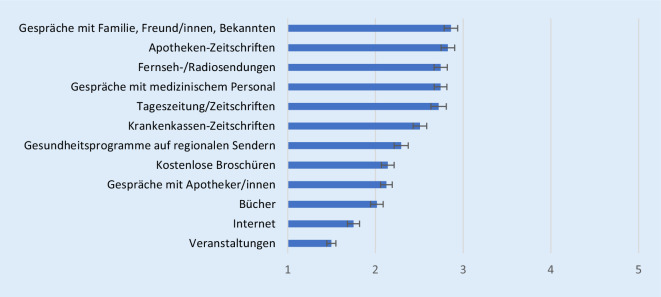

Insgesamt zeigen die Ergebnisse der standardisierten Befragung, dass sich Ältere eher selten bis hin und wieder gezielt über Gesundheit informieren. Innerhalb dieser niedrigen Nutzungshäufigkeit zählen Gespräche, sowohl mit Familie und Freund/innen als auch mit medizinischem Personal, traditionelle Massenmedien wie Fernseh‑/Radiosendungen und Tageszeitungen/Zeitschriften sowie gesundheitsspezifische Angebote wie Apotheken- und Krankenkassenzeitschriften zu den stärker genutzten Quellen (Abb. 1). Das Internet wird hingegen insgesamt eher selten bis gar nicht zur Informationssuche herangezogen.

**Figure 2:**
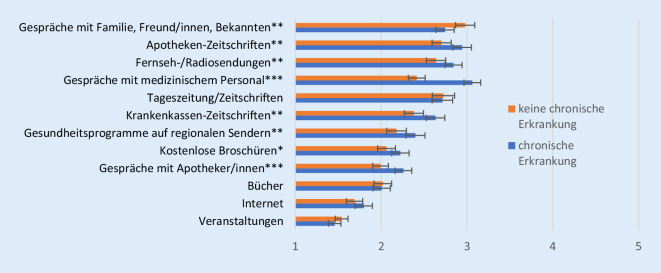

Möchte man verschiedene Gruppen innerhalb der älteren Bevölkerung gezielt erreichen, so sollten Unterschiede im Gesundheitsinformationsverhalten berücksichtigt werden. Ältere mit einer chronischen Erkrankung nutzen gesundheitsspezifische Quellen wie medizinisches und pharmazeutisches Personal, Apotheken- und Krankenkassenzeitschriften sowie Gesundheitsprogramme auf regionalen Sendern signifikant häufiger als jene ohne chronische Erkrankung (Abb. 2). Gespräche mit medizinischem Personal und Apothekenzeitschriften stellen dabei die am häufigsten genutzten Quellen dar.

**Figure 3:**
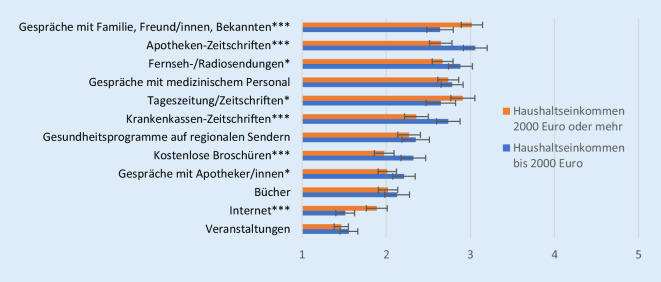

Hinsichtlich des Einkommens lassen sich ebenfalls eine Reihe von Unterschieden in den genutzten Quellen für Gesundheitsinformationen feststellen (Abb. 3). Jene mit höherem Einkommen sprechen häufiger mit dem persönlichen Umfeld über Gesundheit und nutzen häufiger Tageszeitungen/Zeitschriften und das Internet, um sich über Gesundheit zu informieren, als Personen mit niedrigerem Einkommen. Für Ältere mit niedrigerem Haushaltseinkommen stellen hingegen Apothekenzeitschriften und Fernseh‑/Radiosendungen besonders wichtige Gesundheitsinformationsquellen dar. Auch kostenlose Angebote wie Broschüren und Krankenkassenzeitschriften nutzen sie häufiger als Personen mit höherem Einkommen.

**Figure 4:**
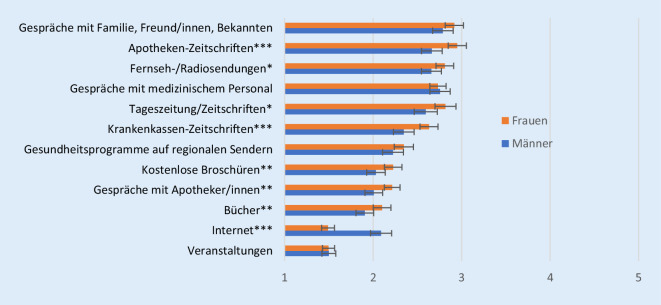

Frauen und Männer unterscheiden sich ebenfalls in der Nutzungshäufigkeit der verschiedenen Quellen (Abb. 4). Die meisten Quellen werden dabei von Frauen häufiger genutzt als von Männern; dies trifft insbesondere für Apothekenzeitschriften zu. Innerhalb der niedrigeren Nutzungshäufigkeit der Männer sind die am meisten genutzten Quellen Gespräche mit persönlichen Kontakten und medizinischem Personal. Weiterhin nutzen Männer deutlich häufiger als Frauen das Internet, um sich über Gesundheit zu informieren.

**Figure 5:**
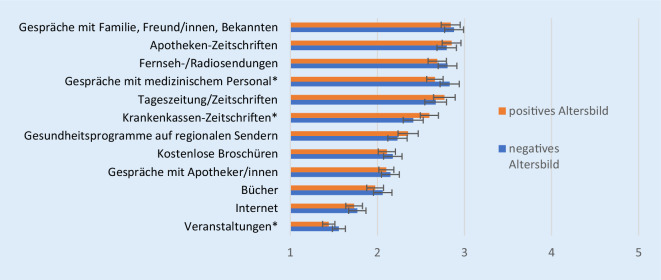

In Bezug auf das Altersbild gibt es nur wenig Unterschiede der genutzten Gesundheitsinformationsquellen (Abb. 5). Personen mit einem eher negativen Altersbild sprechen etwas häufiger mit medizinischem Personal über Gesundheit als jene mit einem positiven Altersbild. Hinsichtlich des Besuchs von Veranstaltungen gibt es zwar einen signifikanten Unterschied, diese Form der Gesundheitsinformation ist jedoch auch bei jenen mit eher negativem Altersbild die am wenigsten genutzte. Relevante Arten der Informationsnutzung für diese Zielgruppe sind hingegen sowohl interpersonale als auch massenmediale Quellen.

## Fazit und Ausblick

Die Ergebnisse der vorliegenden Studie decken sich mit anderen Repräsentativdaten zum Gesundheitsinformationsverhalten der ab 60-Jährigen in Deutschland [12] und zeigen, dass Ältere vorrangig interpersonale Quellen wie medizinisches Personal, Familie und den Freundeskreis sowie Apothekenzeitschriften und andere traditionelle Medienquellen (z. B. TV, Tageszeitung) heranziehen, um sich über Gesundheitsthemen zu informieren. Obwohl die Bedeutung des Internets als Gesundheitsinformationsquelle generell zunimmt [35–37], hat es für ältere Personen einen bisher eher geringen Stellenwert [12].

Insgesamt suchen Ältere eher selten gezielt nach Gesundheitsinformationen. Betrachtet man Daten zu den Alltagsaktivitäten von älteren Bürgerinnen und Bürgern in Deutschland [28], wird jedoch deutlich, dass die Nutzung von Massenmedien wie Zeitungen (insbesondere regionale Tageszeitungen), Zeitschriften, Fernsehen und Radio grundsätzlich einen hohen Stellenwert im Alltag einnimmt. In Verbindung mit der Tatsache, dass Ältere ein eher hohes Interesse an Gesundheit aufweisen, kann die Zielgruppe deshalb über die klassischen Massenmedien vermutlich dennoch gut mit Gesundheitsthemen erreicht werden – auch wenn sie nicht gezielt danach suchen. Hierzu sollte in künftigen Studien neben der aktiven Suche nach Gesundheitsinformationen auch deren passive Aufnahme („information scanning“) berücksichtigt werden. Beide Arten der Informationsaufnahme haben potenziell Einfluss auf gesundheitsbezogene Einstellungen und das Gesundheitsverhalten [38, 39].

Über diesen allgemeinen Befund hinaus müssen Unterschiede zwischen verschiedenen Teilzielgruppen berücksichtigt werden, um diese gezielt über bestimmte Kanäle ansprechen zu können. So informieren sich Frauen insgesamt öfter als Männer über Gesundheit und nutzen hierfür am häufigsten Apothekenzeitschriften. Männer hingegen bevorzugen interpersonale Quellen und nutzen häufiger als Frauen das Internet zur Gesundheitsinformationssuche. Chronisch Erkrankte informieren sich häufig in gesundheitsspezifischen Quellen, insbesondere Gespräche mit medizinischem Personal spielen dabei eine zentrale Rolle. Personen mit niedrigerem Haushaltseinkommen können am besten über Apotheken- und Krankenkassenzeitschriften sowie Radio- und Fernsehsendungen erreicht werden. Dabei wird deutlich, dass sie insbesondere kostenfreie Angebote häufiger nutzen als Personen mit höherem Einkommen. Bezüglich des Altersbildes zeigten sich kaum Unterschiede im Gesundheitsinformationsverhalten. Die Zielgruppe jener mit einem eher negativen Altersbild kann also ebenso wie die Älteren insgesamt sowohl über interpersonale als auch massenmediale Quellen erreicht werden.

Aus Sicht eines Kommunikators können diese Ergebnisse genutzt werden, um gezielt Informationen zu verbreiten: Beispielsweise könnten Informationen über Verfahren zur Früherkennung von Brustkrebs für ältere Frauen mit einem niedrigen sozioökonomischen Status bevorzugt über kostenlose Apothekenzeitschriften distribuiert werden. Dieser Beitrag stellt somit vor allem die Notwendigkeit der Selektion geeigneter Kanäle in den Fokus. Das Beispiel macht außerdem deutlich, dass die in diesem Beitrag getrennt betrachteten Merkmale mitunter in Kombination in die Planung einfließen müssen. So treten manche Merkmale wie ein niedriger sozioökonomischer Status bzw. ein niedriges Einkommen und chronische Erkrankungen häufig gemeinsam auf [18]. Für beide Merkmale zeigte sich, dass auch in diesem Fall Apothekenzeitschriften eine bevorzugte Quelle sind, da dieses Medium sowohl kostenlos ist als auch ein gesundheitsspezifisches Angebot darstellt.

Mit Blick auf die theorie- und evidenzbasierte Planung strategischer Gesundheitskommunikation [7] müssen jedoch neben der Auswahl geeigneter Quellen auch die Inhalte und die Gestaltung von Kommunikationsmitteln auf die Zielgruppe angepasst werden. Bei der Aufbereitung von Materialien für eine ältere Zielgruppe muss beispielsweise berücksichtigt werden, dass im Alter kognitive und körperliche Fähigkeiten abnehmen [40] und es Älteren schwerfällt, komplexe Situationen zu verarbeiten und die Relevanz verschiedener Informationen zu differenzieren [41]. Darüber hinaus sollten neben der Auswahl geeigneter Quellen auch die konkreten Inhalte der Kommunikationsmaßnahmen evidenzbasiert für das jeweilige Gesundheitsverhalten formuliert werden (für ein Beispiel siehe [28] zu körperlicher Aktivität bei Älteren).

## Footnotes

1 Das Projekt wurde unterstützt durch die Bundeszentrale für gesundheitliche Aufklärung (BZgA) im Rahmen von *Älter werden in Balance*, einem Programm mit finanzieller Unterstützung des Verbandes der Privaten Krankenversicherung e. V. (PKV). Die vollständigen Ergebnisse des Projekts können im Abschlussbericht nachgelesen werden [28].

2 Die Formulierungen der Befragten wurden im Wortlaut übernommen und grammatikalische Fehler beibehalten.
